# Supplementary material for: Poor sleep quality and suicidal ideation among pregnant women during COVID-19 in Ethiopia: systematic review and meta-analysis
Source: PeerJ. 2023 Sep 29;11:e16038. doi: 10.7717/peerj.16038 (PMC10544305; doi:10.7717/peerj.16038)
Supplement: Supplemental Information 2 [file peerj-11-16038-s002.docx]

1. **The rationale for conducting the systematic review and meta-analysis;**

COVID-19 pandemic has impacted mental health and well-being of pregnant women worldwide. Studies in Ethiopia reported that due to COVID-19 pandemic poor sleep quality and suicidal ideation were increased. This study proposed to assess the prevalence and associated factors of poor sleep quality and suicidal ideation among pregnant women during COVID-19 in Ethiopia. No study reported in a summarized way. So, a thorough investigation is necessary, as is the provision of thorough evidence regarding the prevalence and contributing variables of poor sleep quality and suicidal ideation among pregnant women in Ethiopia is crucial.Measuring the combined prevalence of poor sleep quality, suicidal ideation, and their associated factors is the main aim. This study offers proof of mental health issues in pregnant women to academics and decision-makers.

1. **The contribution that it makes to knowledge in light of previously published related reports, including other meta-analyses and systematic reviews;**

COVID-19 pandemic highly affects the mental health of pregnant women. Worldwide meta-analysis result showed that the prevalence of poor sleep quality and suicidal ideation were increased due to this pandemic. Also studies in Ethiopia showed the prevalence of poor sleep quality and suicidal ideation were increased. This study provides comprehensive evidence on prevalence and associated factors of poor sleep quality and suicidal ideation among pregnant women during COVID-19 pandemic in Ethiopia.
