# Supplementary material for: Poor sleep quality and suicidal ideation among pregnant women during COVID-19 in Ethiopia: systematic review and meta-analysis
Source: PeerJ. 2023 Sep 29;11:e16038. doi: 10.7717/peerj.16038 (PMC10544305; doi:10.7717/peerj.16038)
Supplement: Supplemental Information 3 [file peerj-11-16038-s003.docx]

**Supplementary file 1**: Search strategy for PubMed, Google Scholar and African Journals Online databases

| **Databases** | **Search number** | **Search detail** |
| --- | --- | --- |
| 1. **PubMed** | #1 | “COVID-19”[MeSH Terms] |
|  | #2 | “mental disorder”[Mesh Terms] |
|  | #3 | “pregnancy” [Mesh Terms] |
|  | #4 | “COVID-19”[Title/Abstract] OR “2019 novel coronavirus disease”[Title/Abstract] OR “2019 novel coronavirus infection”[Title/Abstract] OR “2019 ncov disease”[Title/Abstract] OR “2019 ncov infection”[Title/Abstract] OR “covid 19 pandemic”[Title/Abstract] OR “covid 19 pandemics” [Title/Abstract] OR “covid 19 virus disease”[Title/Abstract] OR “covid 19 virus infection”[Title/Abstract] OR “COVID19”[Title/Abstract] OR “coronavirus disease 2019”[Title/Abstract] OR “coronavirus disease 19”[Title/Abstract] OR “sars coronavirus 2 infection”[Title/Abstract] OR “sars cov 2 infection”[Title/Abstract] OR “severe acute respiratory syndrome coronavirus 2 infection” [Title/Abstract] OR “SARS-CoV-2” [Title/Abstract] OR “2019 novel coronavirus” [Title/Abstract] OR “2019 novel coronavirus”[Title/Abstract] OR “2019- nCoV”[Title/Abstract] OR “covid 19 virus”[Title/Abstract] OR “covid19 virus”[Title/Abstract] OR  “Coronavirus disease 2019 virus”[Title/Abstract] OR “SARS coronavirus 2” [Title/Abstract] OR “SARS cov 2 virus”[Title/Abstract] OR “severe acute respiratory syndrome coronavirus 2”[Title/Abstract] OR “Wuhan coronavirus”[Title/Abstract] OR “Wuhan seafood market pneumonia virus”[Title/Abstract] |
|  | #5 | “mental illness” [Title/Abstract] OR “psychiatric problem” [Title/Abstract] OR “psychology problem” [Title/Abstract] OR “mental health effect” [Title/Abstract] OR “psychological disturbance” [Title/Abstract] OR “mental disorder” [Title/Abstract] OR “psychiatric illness” [Title/Abstract] OR “psychiatric diseases” [Title/Abstract] OR “psychiatric disorders” [Title/Abstract] OR “behavior disorders” [Title/Abstract] OR “severe mental disorder” [Title/Abstract] OR “sleeping quality” [Title/Abstract] OR “poor sleep quality” [Title/Abstract] OR “insomnia” [Title/Abstract] AND “suicide” [Title/Abstract] OR “suicidal ideations” [Title/Abstract] |
|  | #6 | “pregnant women”[Title/Abstract] OR “prenatal” [Title/Abstract] perinatal” OR “postpartum” [Title/Abstract] OR “antenatal” [Title/Abstract] OR “postnatal” [Title/Abstract] OR “peurperal” [Title/Abstract] OR “lactating women” [Title/Abstract] OR “reproductive age women” [Title/Abstract] OR “child bearing women” [Title/Abstract] AND “Ethiopia”[Title/Abstract] |
|  | #7 | #1 OR #4 |
|  | #8 | #2 OR #5 |
|  | #9 | #3 OR #6 |
|  | #10 | #7 AND #8 AND #9 |
|  | #11 | Limit to observational study” OR “cross-sectional” |
| 1. Google Scholar and African Journals Online | #1 | COVID-19 OR coronavirus disease 2019 OR SARS CoV-2 OR 2019 novel coronavirus disease OR 2019 novel coronavirus infection OR 2019 ncov disease OR 2019 ncov infection OR covid 19 pandemic OR covid 19 pandemics OR covid 19 virus disease OR covid 19 virus infection OR COVID19 OR coronavirus disease 19 OR sars coronavirus 2 infection OR sars cov 2 infection OR severe acute respiratory syndrome coronavirus 2 infection OR 2019 novel coronavirus OR 2019 novel coronavirus |
|  | #2 | “mental illness” OR “psychiatric problem” OR “psychology problem” “mental health effect” OR “psychological disturbance” OR “mental disorder” OR “psychiatric illness” OR “psychiatric diseases” OR “psychiatric disorders” OR “behavior disorders” OR “severe mental disorder” OR “sleeping quality” OR “poor sleep quality” OR “insomnia” AND “suicide” |
|  | #3 | “pregnant women” OR “prenatal” perinatal” OR “postpartum” OR “antenatal” OR “postnatal” OR “peurperal” OR “lactating women” OR “reproductive age women” OR “child bearing women” AND “Ethiopia” |
|  | #4 | #1 AND #2 AND #3 |
|  | #5 | Limit to studies published till the end of February 2023 |
|  | #6 | Limit to Humans |
|  | #7 | Limit to observational study OR cross-sectional |
